# Development and evaluation of a culturally adapted digital-platform integrated multifaceted intervention to promote the utilization of maternal healthcare services: a single-arm pilot study

**DOI:** 10.1186/s12939-023-02033-y

**Published:** 2023-10-17

**Authors:** Jiayao Xu, Hailati Akezhuoli, Meng Zhou, Tingting Yao, Jingjing Lu, Xiaomin Wang, Xudong Zhou

**Affiliations:** 1 Institute of Social Medicine, School of Medicine, Zhejiang University, 866 Yuhangtang Road, Xihu District, Hangzhou, 310058 China; 2 Sir Run Run Shaw Hospital, Zhejiang University School of Medicine, Hangzhou, China; 3 School of Public Health, Hangzhou Normal University, No. 2318 Yuhangtang Road, Yuhang District, Hangzhou, 311121 China; 4 The Second Affiliated Hospital, Zhejiang University School of Medicine, 88 Jiefang Road, Hangzhou, 310009 China

**Keywords:** Maternal healthcare, Ethnic minority, Digital health, Health education

## Abstract

**Background:**

The utilization of hospital delivery and antenatal care (ANC) is essential for improving maternal and newborn outcomes. However, social and cultural barriers in underdeveloped rural areas hindered maternal care utilization. This study aims to design and evaluate the effectiveness of a culturally adapted digital-platform intervention to promote maternal care utilization among women in ethnic minority communities in China.

**Methods:**

From January 1st, 2020, to December 31st, 2021, all pregnant women in Mianshan town, Liangshan Autonomous Prefecture, were invited to participate in the intervention. The multifaceted intervention included participatory and cultural-tailored health education on a popular social media platform, transportation subsidies, and capacity building and economic incentives for healthcare providers. The effectiveness of the intervention was evaluated by comparing two groups: mothers who gave live birth before the intervention (January 1st to December 31st, 2019) and mothers whose entire pregnancy period was covered by the intervention. The primary outcomes were the rate of hospital delivery and ANC utilization. Data on pregnant women were retrospectively collected through telephone surveys and the maternal and newborn’s health monitoring system.

**Results:**

A total of 237 intervention sample and 138 pre-intervention sample were included. The intervention group demonstrated significantly higher rates of hospital delivery (97.5% vs. 87.7%, p < 0.001), timely initiation of ANC (73.0% vs. 62.3%, p = 0.031), and timely completion of five-time ANC visits (37.1% vs.4.3%, p < 0.001) compared to the pre-intervention group. The intervention group was more likely to utilize hospital delivery (OR = 9.26, 95%CI [2.83–30.24], p < 0.001) and ANC, including timely initiation of ANC (OR = 2.18, 95%CI [1.31–3.62], p = 0.003), completion of five ANC visits (OR = 1.72, 95%CI [1.05–2.83], p = 0.032), and timely completion of five ANC visits (OR = 15.12, 95%CI [6.24–36.64], p < 0.001).

**Conclusions:**

The culturally adapted digital-platform integrated multifaceted intervention effectively promoted the utilization of hospital delivery, timely initiation of ANC, and completion of ANC visits in the Yi ethnic community in China. This study provides valuable insights for future interventions targeting maternal healthcare services in underdeveloped ethnic minority communities worldwide.

**Trial registration:**

Chinese Clinical Trial Registry, ChiCTR2300073219. Registered 4 July 2023 - Retrospectively registered, https://www.chictr.org.cn/showproj.html?proj=199202.

**Supplementary Information:**

The online version contains supplementary material available at 10.1186/s12939-023-02033-y.

## Background

Despite significant progress in reducing global maternal mortality rates over the decades, the current levels remain unacceptably high [1]. An estimated 292,000 women died globally in 2020 due to preventable causes related to pregnancy and childbirth, with the majority of these deaths occurring in low-resource settings [2]. Access to high-quality antenatal care (ANC) and hospital delivery, supported by skilled health personnel, is crucial for improving survival rates among both mothers and babies [3, 4]. However, only 66% of pregnant women received the minimum of four contacts for ANC visits recommended by the World Health Organization (WHO) globally from 2015 to 2021 [5]. There is an urgent need for intensified efforts to improve the utilization of maternal healthcare services among women in low-resource settings.

The Chinese government initiated a program offering free ANC and subsidized hospital delivery in 2010 [6]. Pregnant women were advised to have their first ANC visit by the 13th week of gestation, with subsequent visits recommended during the 16–20, 21–24, 28–36, and 37–40 week periods, respectively [7]. Despite notable progress, disparities in access to and utilization of antenatal and hospital delivery care persist, particularly among women in disadvantaged areas. Liangshan Autonomous Prefecture, also known as Liangshan, is one of the most underdeveloped regions in Sichuan province in western China, characterized by a multi-ethnic population with 54.56% Yi ethnicity [8]. According to the National Health Service Surveys in Sichuan province, pregnant women in ethnic minority counties, including Liangshan, reported lower rates of ANC and hospital deliveries compared to those in non-minority districts and counties [9]. This disparity was particularly pronounced among ethnic minority women residing in ethnic minority counties [9]. It was estimated that the difference in hospital delivery rates between ethnic minority counties and non-minority counties was responsible for 74.5% of the maternal mortality disparity in Sichuan province [10]. Previous studies in Liangshan have identified several barriers to the utilization of antenatal and hospital delivery care, including women’s low education levels [11, 12], poor awareness of ANC and hospital delivery [11], women’s subordinate position within their own household [12], the traditional norm of delivery at home [11], poor affordability of transportation costs for accessing healthcare facilities [12], and low quality of healthcare services [13]. It is important to note that the barriers mentioned above are common challenges in disadvantaged areas worldwide. Effective interventions aimed at addressing these barriers will be of great significance.

With the widespread adoption of mobile phones globally, digital health interventions have demonstrated great potential in providing health education, advice delivery, and appointment reminders to improve access to maternal and childbirth care. Researchers have implemented various digital-platform-based interventions in low-resource settings to promote the completion of ANC check-ups and facility-based deliveries, utilizing methods such as phone calls, text messaging, and animated films [14–16]. Digital interventions hold significant promise in underserved regions due to their low cost and potential to empower vulnerable women, supporting their decision-making processes. The social and cultural characteristics of the local society were considered to effectively integrate digital technologies into existing healthcare systems and to maximize the potential of the multifaceted intervention.

This study aimed to conduct a multifaceted intervention and adopt WeChat group chat-based health education to address barriers to ANC utilization and hospital delivery in a Yi ethnic minority community in China, and to evaluate the potential effects of the interventions.

## Methods

### Study area

This pilot study was conducted in a predominantly mountainous rural town, Mianshan town, located in Xide County, Liangshan, Sichuan Province. Mianshan town had more than 15 thousand residents, with 91.49% being of the Yi Minority [17, 18]. It had one township hospital and one rural doctor in each village. ANC services in Mianshan town are offered by maternal personnel in the township hospital and delivery services are provided by county hospitals. The village doctors play a role in reminding pregnant women to complete their ANC visits on time.

### Intervention design

**Fig. 1:**
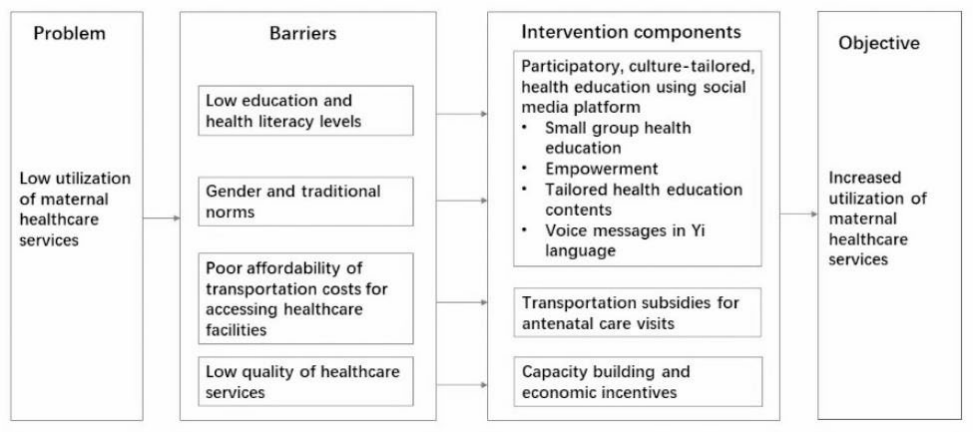
Framework of the intervention design

We designed a multifaceted intervention to encourage timely and adequate ANC utilization and hospital delivery among pregnant women in Mianshan Town. In-depth face-to-face stakeholder interviews were conducted among 73 pregnant women (69 of Yi ethnic, 60 with primary school or under education level), 5 maternal care health workers from the township hospital, and 11 from the county hospitals. The detailed sociodemographic characteristics of the participants in the interviews are described in the supplementary tables (See Additional file 1, Table S1-S2). In the qualitative study, we identified several barriers to the utilization of maternal healthcare services, including (1) low education and health literacy levels among the targeted population; (2) outdated gender and traditional norms; (3) poor affordability of transportation costs for accessing healthcare facilities; (4) low quality of healthcare services. Tailored multifaceted interventions were developed to address these barriers (Fig. 1).

### Participatory health education

A participatory health education intervention was conducted utilizing WeChat, a widely used free social media and messaging platform in China. We used WeChat group chats for health education among the targeted population. Pregnant women in the same gestational month were invited to join the same WeChat group chat as they share similar health education and management needs. Seven small WeChat group chats, each consisting of 20–30 pregnant women in the same gestational month, were simultaneously established. Within each group chat, an ANC provider from the township hospital was readily available to communicate with pregnant women. A young and open-minded mother was recruited as a volunteer to assist the ANC provider in each group. Tailored health education was designed to address the barriers including essential topics, such as the hospital delivery subsidy policy, the significance of hospital delivery, the importance of ANC (e.g., B ultrasound), the appropriate timing and frequency of ANC, and essential precautions during pregnancy. The health education materials were imbued with elements derived from Yi ethnic culture, such as totems, fostering cultural sensitivity and relevance [11, 19]. The Yi ethnic group has its own spoken language. Health education materials in audio or voice formats were presented in the Yi language within the WeChat group chats. All audio health education materials were delivered in the Yi language, and textual materials were explained verbally using voice messages in the Yi language. This approach ensured that even illiterate pregnant women could readily comprehend the content being conveyed.

### Transportation subsidies for pregnant women

Limited family income often hinders the ability to afford the transportation costs required to access healthcare facilities. Due to the mountainous terrain and an underdeveloped transportation network in Liangshan, visits to the township and county-level hospitals can be costly and financially burdensome, particularly for residents residing in remote mountainous areas. Recognizing this challenge, the government has been offering transportation subsidies to assist with the costs associated with hospital deliveries since 2009 [20]. We provided transportation subsidies for pregnant women’s ANC attendance to facilitate their completion of ANC visits. The allocation of transportation subsidies for ANC visits is based on the distance between their home and the township hospital.

### Capacity building and economic incentives for healthcare providers

Inadequate capacity and low incentives of healthcare providers hinder the provision of quality maternal healthcare services. A short-term small-class training course, which covered essential aspects of ANC, including B ultrasound, and blood routine examinations, was designed and conducted for ANC providers. Village doctors played a crucial role in notifying pregnant women about the importance of timely utilization of maternal healthcare services. A monetary reward for village doctors of 40 RMB for each timely initiation of an ANC visit and 10 RMB for each completed ANC visit were introduced.

### Study design and evaluation

**Fig. 2:**
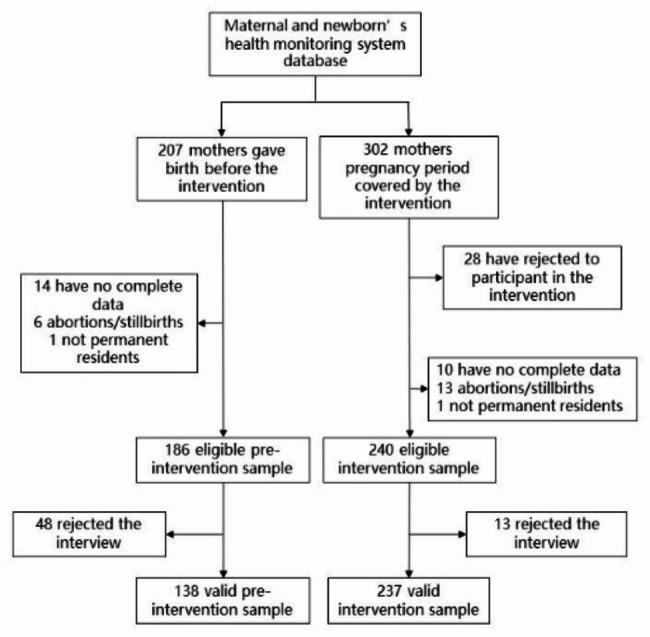
Flow of participants in the intervention evaluation

A single-arm pilot intervention was conducted in Mianshan town from January 1st, 2020, to December 31st, 2021, targeting all pregnant women during this period in Mianshan town. Pregnant women 16 years old or above were invited to take part in the intervention. The intervention was evaluated by comparing the differences in maternal healthcare utilization among women who gave live birth before the intervention (January 1st to December 31st, 2019) and women who were pregnant after January 1st, 2020 and gave live birth during the intervention (January 1st, 2020, to December 31st, 2021). Women who gave live birth during the intervention but were already pregnant before January 1st, 2020, were excluded from the intervention evaluation because the entire pregnancy period for these women was not covered by the intervention. Women who were not-local residents or those who were residents but had resided in Mianshan town for less than six months, and those with missing complete ANC data were excluded. Village doctors were responsible for inviting eligible pregnant women to participate in the intervention evaluation (Fig. 2). A list of eligible pregnant women with telephone numbers was provided by the township hospital ANC providers. The village doctors were trained to conduct telephone or face-to-face interviews to collect data.

### Data collection

The questionnaire and the maternal and newborn’s health monitoring system were utilized to collect data for evaluation. The primary outcomes are the rate of maternal care utilization, including the rate of hospital delivery and the ANC utilization. Participants were asked about their delivery places of the last birth (hospital delivery/ non-hospital delivery). The ANC utilization, including timely ANC initiation, completion of five-time visits, and timely completion of five-time visits, was collected from the maternal and newborn’s health monitoring system. The secondary outcomes are the intervention sample’s involvement in the intervention (e.g., receiving transportation subsidies, participation in health education on group chats), which were collected by the interview. Sociodemographic information including pregnant women’s ethnicity (Yi/ Han), parity, education levels (Illiterate/ Primary school/ Junior high school or above), age (< 20 years / 20–25 years/ 26–30 years/ >30 years), annual household income (≤ 5000 RMB/ 5001–10,000 RMB/ ≥10,001 RMB), rural-urban migration experience (Yes/ No), and walking time from home to the township hospital (< 30 min/ 30–45 min/ 46–60 min/ >60 min) were recorded. All participants provided written informed consent, and for those unable to write, a thumbprint was affixed to the consent form. Our study was reviewed and approved by the Ethics Committee of the School of Public Health at Zhejiang University (No. ZGL201906-4).

### Data analysis

Thematic analysis was applied to the qualitative data. Descriptive analyses were conducted to show the proportions and frequencies in the quantitative data. The differences in maternal care utilization and sociodemographic characteristics between the pre-intervention and intervention group were assessed using the chi-squared test and Fisher’s exact test. Multivariable logistic regressions were employed to evaluate differences in the primary outcomes (i.e., hospital delivery and ANC utilization) between the two groups. The pre-intervention group was used as the reference, and adjustments were made to sociodemographic information. Based on previous literature [9, 21], we identified sociodemographic characteristics associated with maternal care utilization. These include ethnicity, education levels, annual household income, rural-urban migration experience, parity, and walking time from home to the township hospital. Consequently, we adjusted for these sociodemographic characteristics in our analysis. Additionally, a secondary analysis was performed to assess the correlation between participation levels in the intervention (i.e., whether participants received transportation subsidies for antenatal care visits, joined the WeChat group chat, browsed health education information in the WeChat group chat, understood health education information, or interacted in the WeChat group chat) and maternal care utilization, aiming to explore the potential effects of the specific intervention components among the intervention sample. Furthermore, sociodemographic characteristics and levels of participation in the intervention were also compared. All statistical analyses were performed using SPSS 24.0 with the statistical significance set at p < 0.05.

## Results

In total, 237 women were included in the intervention group, and 138 women were included in the pre-intervention group in the intervention evaluation survey. Table 1 presents the maternal care utilization and sociodemographic characteristics across the groups. No significant differences were observed between the two groups in terms of education, age, annual household income, rural-urban migration experience, or walking time from home to the township hospital. The intervention group had a slightly higher proportion of Yi ethnicity (97.5% vs. 92.8%, p = 0.029) and a higher number of childbirths (p = 0.016) than the pre-intervention group. The intervention group reported significantly higher rates of hospital delivery (97.5% vs. 87.7%, p < 0.001), timely initiation of ANC (73.0% vs. 62.3%, p = 0.031), and timely completion of five-time ANC visits (37.1% vs.4.3%, p < 0.001) than the pre-intervention group. The intervention group demonstrated a higher rate of completion of five-time visits (77.2% vs. 68.1%, p = 0.053) with a marginal significance.

**Table 1:** Associations between intervention and maternal healthcare utilization among the participants (N = 375)

	Pre-intervention group (n = 138)	Intervention group (n = 237)	p
**Primary outcomes**			
Hospital delivery	121(87.7)	231(97.5)	< 0.001
Timely initiation of antenatal care	86(62.3)	173(73.0)	0.031
Completion of five antenatal care visits	94(68.1)	183(77.2)	0.053
Timely completion of five antenatal care visits	6(4.3)	88(37.1)	< 0.001
**Sociodemographic characteristics**			
**Ethnicity**			0.029
Han	10(7.2)	6(2.5)	
Yi	128(92.8)	231(97.5)	
**Education**			0.815
Illiterate	26(18.8)	40(16.9)	
Primary school	81(58.7)	138(58.2)	
Junior high school or above	31(22.5)	59(24.9)	
**Age**			0.490
< 20	25(18.1)	30(12.7)	
20–25	59(42.8)	115(48.5)	
26–30	34(24.6)	59(24.9)	
> 30	20(14.5)	33(13.9)	
**Annual household income**			0.346
≤ 5000 RMB	31(22.5)	47(19.8)	
5001–10,000 RMB	58(42.0)	118(49.8)	
≥ 10,001 RMB	49(35.5)	72(30.4)	
**Rural-urban migration experience**			0.680
No	25(18.1)	39(16.5)	
Yes	113(81.9)	198(83.5)	
**Parity**			0.016
1	59(42.8)	72(30.4)	
2	38(27.5)	77(32.5)	
3	31(22.5)	49(20.7)	
≥ 4	10(7.2)	39(16.5)	
**Walking time from home to the township hospital**			0.357
< 30 min	16(15.0)	30(12.7)	
30-45 min	30(28.0)	60(25.3)	
46-60 min	37(34.6)	101(42.6)	
> 60 min	24(22.4)	46(19.4)	

**Fig. 3:**
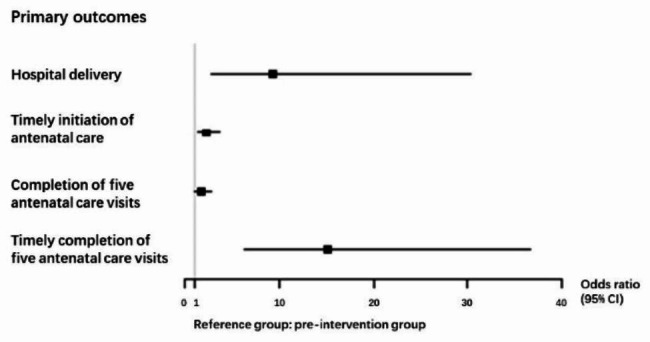
Forest plot for the intervention effects

The intervention group were more likely to give birth in a hospital (Odds ratio [OR] = 9.26,95%CI [2.83–30.24], p < 0.001) compared to the pre-intervention group after adjusting ethnicity, education, annual household income, rural-urban migration experience, parity, and walking time from home to the township hospital (Table 2; Fig. 3). Meanwhile, the intervention group showed an increased likelihood of completion of the ANC, including timely initiation of ANC (OR = 2.18,95%CI [1.31–3.62], p = 0.003), completion of five-time visits (OR = 1.72,95%CI [1.05–2.83], p = 0.032), and timely completion of five-time visits (OR = 15.12,95%CI [6.24–36.64], p < 0.001) throughout their pregnancy.

**Table 2:** Logistic multivariate models for maternal healthcare services utilization among the participants before and during the intervention (N = 375)

	Odds ratio (95% CI)^a^	p
**Primary outcomes**		
Hospital delivery	9.26(2.83,30.24)	< 0.001
Timely initiation of antenatal care	2.18(1.31,3.62)	0.003
Completion of five antenatal care visits	1.72(1.05,2.83)	0.032
Timely completion of five antenatal care visits	15.12(6.24,36.64)	< 0.001

^a^ Reference group: pre-intervention group; adjusted for ethnicity, education, annual household income, rural-urban migration experience, parity, and walking time from home to the township hospital

Among the intervention group, participants who received transportation subsidies reported a higher rate of hospital delivery (98.6% vs. 88.0%, p = 0.001) (See Additional file 1, Table S3). Participants who joined the WeChat group chat had higher rates of hospital delivery (98.6% vs. 87.0%, p = 0.001) and timely initiation of ANC (75.2% vs. 52.2%, p = 0.018). Participants who browsed health education information in the group chat demonstrated higher rates of timely initiation of ANC (78.5% vs. 66.4%, p = 0.037) and timely completion of five-time visits (43.8% vs. 29.0%, p = 0.018). Participants who reported understanding health education information in the group showed a higher rate of timely initiation of ANC (77.2% vs. 58.5%, p = 0.007). There were no significant differences in the rates of antenatal and hospital delivery care utilization between participants who interacted in the group chat and who did not. The associations between specific intervention methods and sociodemographic characteristics in the intervention group were summarized in the supplementary table (See Additional file 1, Table S4).

## Discussion

In Mianshan town, a multifaceted intervention has been shown to significantly improve maternal care utilization, including increased rates of hospital delivery, timely initiation of ANC, completion of five ANC visits, and timely completion of five ANC visits. This intervention considers the interests of both the service demanders and the service providers to maximize effectiveness [22]. The service users (i.e., pregnant women) were actively involved in the intervention design process, ensuring that their specific needs, Yi ethnic culture, and social background were adequately addressed. The service providers were supported by skill training and economic incentives. The findings of this study highlight the effectiveness of a culturally adapted, digital-platform integrated intervention, which has the potential to be scaled up and promote the utilization of maternal healthcare services in ethnic minority communities in China.

Timely initiation and completion of ANC play a crucial role in promoting hospital delivery and reducing non-hospital delivery, thereby minimizing the risk of life-threatening complications during labor and the postpartum period for both mothers and newborns. In this intervention, several methods were implemented to encourage the timely initiation and completion of ANC. These methods included providing health education within WeChat group chats to emphasize the importance of ANC, its appropriate timing, and frequency. Additionally, transportation subsidies were offered for ANC visits, and economic incentives were provided to village doctors to ensure pregnant women’s timely initiation and completion of ANC visits. The intervention was facilitated by the involvement of village doctors who maintain regular contact and familiarity with pregnant women. This multifaceted intervention successfully encouraged pregnant women’s timely initiation and completion of ANC, and was found to be effective in promoting hospital delivery. Previous research conducted in developing areas demonstrated that women who receive timely and adequate ANC were 2.4–6.8 times more likely to deliver at a healthcare facility compared to those who did not receive ANC [23–26]. During ANC visits, ANC providers offer advice based on maternal and fetal health, facilitating informed decisions regarding childbirth delivery. Moreover, initiating ANC within the first trimester is crucial for establishing a precise pregnancy due date. Based on our qualitative study, some pregnant women experienced on-the-way births to the hospital due to imprecise pregnancy due dates caused by delayed ANC initiation, compounded by transportation challenges. In addition to potential health education during ANC visits, offering health education on WeChat regarding the importance of hospital delivery and the hospital delivery subsidy policy may contribute to a higher hospital delivery rate among the intervention group. This pilot study showed a significant increase of 11.2% in the rate of hospital delivery, accompanied by a 17.2% increase in the rate of timely initiation and a 13.4% increase in the rate of completing five ANC visits.

Digital health was recommended by WHO as a promoter of health utilization [27]. m-Health intervention is accessible, unlimited by distance, cost-effective, and holds promise in improving health equity [28]. Our study echoes WHO by proving the effectiveness of digital-platform-based intervention among Yi women. The intervention group may have been affected by the novel coronavirus disease (COVID-19) pandemic. During the COVID-19 pandemic, social interactions were restricted, and most interactions took place online via digital devices rather than in person [29]. Utilizing social media platforms for health education offered clear advantages in this context. By using the most popular social media WeChat and adopting a multifaceted approach, we promoted maternal healthcare services utilization in the Yi community, even during the pandemic. This social-media-based intervention conducted in a developing ethnic minority community in China offers implications for interventions in developing areas worldwide.

Socially and culturally adapted health education is superior to conventional one, particularly when addressing the unique needs of diverse racial and ethnic populations [30, 31]. In this study, we implemented a user-oriented approach to health education. To effectively meet the unique health education needs of pregnant women at different gestational stages, they were grouped into different health education groups within WeChat. To effectively convey health information regardless of the audience’s education and health literacy levels, we developed multimedia health education materials comprising still images, animations, film footage, and audio. The use of non-textual health education materials proved to be more accessible and accommodating for ethnic populations and individuals with limited education or health literacy levels. Health education materials were carefully tailored to ensure linguistic and cultural appropriateness, effectively capturing the lived experiences of the local population within the ethnic minority community. These culturally sensitive materials incorporate relevant examples, analogies, and stories that deeply resonate with the local culture, while also using names and figures that reflect the community’s language and appearance. The selection of health education themes and topics was carefully curated based on valuable suggestions provided by the targeted population.

Gender inequity in Liangshan has been a long historical problem [32]. Women had limited access to health information and were not able to control household resources. However, we adopted a women’s empowerment approach [33] that fostered mutual support and encouraged active participation among women in the intervention to improve their access to and utilization of health resources. Empowerment can serve as a valuable facilitative approach to addressing the healthcare needs of individuals from disadvantaged social backgrounds. Many cultures, including the Yi culture, adhere to the traditional belief that “men and women should not have intimate contact without formal relationships.“ As local gender norms might discourage women from seeking healthcare services, further studies need to pay attention to the proportion of female healthcare workers in Liangshan healthcare facilities.

This study has several limitations. Firstly, we employed a single-arm study design and evaluated the effects of the intervention based on the comparison between the historical control group and the intervention group, which prevents us from completely isolating the influence of other social changes. For example, social distancing measures during the COVID-19 pandemic may have reduced the utilization of maternal healthcare services during the intervention, potentially underestimating the true intervention effects. Secondly, in addition to data recorded in the maternal and newborn’s health monitoring system, self-reported information about participants’ last birth was collected retrospectively, which could introduce recall bias and social desirability. However, village doctors, who have a close relationship with the villagers, may have helped correct the information by referring to their routine working documents during data collection. We did not collect information on participants’ pregnancy outcomes (e.g., preterm deliveries, pregnancy complications), which could have provided additional insights into the impact of our intervention. Thirdly, assessing the improvement of participants’ knowledge and awareness regarding maternal care utilization was challenging due to their high illiteracy rate. This limitation hindered our ability to fully capture the process of intervention effects. However, health education materials were delivered in audio or voice formats in the Yi language to ensure equal accessibility for both literate and illiterate women. Furthermore, women in Liangshan endured a long history of gender inequity, and the decision of whether to give birth at home or in hospitals is typically made by family members, particularly mothers-in-law and husbands, rather than the pregnant women themselves. Unfortunately, we did not include intervention elements targeting mothers-in-law and husbands, but it deserves further investigation. Lastly, it should be noted that this pilot study was conducted in Mianshan town, Xide county, within Liangshan—an underdeveloped rural area with its own unique cultural and social context. Therefore, the generalizability of this intervention to a broader population remains uncertain.

## Conclusions

A culturally adapted multifaceted intervention, based on the township hospital and targeting both healthcare providers and healthcare users, has proven to be effective in promoting the utilization of maternal healthcare services in underdeveloped rural areas with a Yi ethnic culture in China. This study adds further evidence supporting the promotion of maternal healthcare services, which in turn contributes to improved maternal and newborn outcomes. Social-media-based health education demonstrates its potential in promoting health equity in developing areas.

### Electronic supplementary material

Below is the link to the electronic supplementary material.

Supplementary Material

## Data Availability

The datasets used and/or analysed during the current study are available from the corresponding author on reasonable request.

## Abbreviations

ANC: Antenatal care
WHO: World Health Organization
