# Supplementary material for: Development and evaluation of a culturally adapted digital-platform integrated multifaceted intervention to promote the utilization of maternal healthcare services: a single-arm pilot study
Source: Int J Equity Health. 2023 Oct 17;22:217. doi: 10.1186/s12939-023-02033-y (PMC10583400; doi:10.1186/s12939-023-02033-y)
Supplement: Supplementary file 1 — Supplementary Material [file 12939_2023_2033_MOESM1_ESM.docx]

**Table S1** **Sociodemographic characteristics of the pregnant women in the qualitative interviews**

|  | **County**  **(n=11)** | **Township (n=5)** |
| --- | --- | --- |
| **Sex** |  |  |
| Male | 0(0.0) | 2(40.0) |
| Female | 11(100.0) | 3(60.0) |
| **Age, ‾X (SD)** | 37(9.4) | 30.6(6.0) |
| **Education** |  |  |
| Below Bachelor’s degree | 1(9.1) | 1(20.0) |
| Bachelor’s degree and above | 10(90.9) | 4(80.0) |
| **Job professional level** |  |  |
| None | 2(18.2) | 2(40.0) |
| Entry level | 3(27.3) | 3(60.0) |
| Intermediate level | 5(45.5) | 0(0.0) |
| Senior level | 1(9.1) | 0(0.0) |
| **Department** |  |  |
| Pediatrics | 2(18.2) |  |
| Obstetrics and gynecology | 7(63.6) |  |
| Management | 2(18.2) | 2(40.0) |
| Maternal and child community health worker |  | 3(60.0) |

**Table S2** **Sociodemographic characteristics of the maternal care health workers in the qualitative interviews**

| **Sociodemographic characteristics** | **(n=73)** |
| --- | --- |
| **Ethnicity** |  |
| Yi | 69(94.5) |
| Others | 4(5.5) |
| **Age, ‾X (SD)** | 24.7(4.3) |
| **Education completed** |  |
| Illiterate | 28(38.3) |
| Some primary school | 15(20.5) |
| Primary school | 17(23.3) |
| Middle school | 9(12.3) |
| High school/secondary school | 3(4.1) |
| College | 1(1.4) |
| **Profession** |  |
| Unemployed/farmer | 59(80.8) |
| Teacher/company employee | 24.7(4.3) |
| Migrant workers | 9(12.3) |
| Self-employed | 3(4.1) |
| **Annual household income,** |  |
| ≤5000 RMB | 16(22.2) |
| 5001-10000 RMB | 17(23.6) |
| 10001-20000 RMB | 13(18.1) |
| 20001-30000 RMB | 7(9.7) |
| 30001-40000 RMB | 6(8.3) |
| 40001-50000 RMB | 4(5.6) |
| >50000 RMB | 6(8.3) |

**Table S3 Associations between specific intervention methods and maternal healthcare utilization in Intervention group (n=237)**

|  | **Total** | **Delivery places** | | **Completion rate of antenatal care visits** | | | | | |
| --- | --- | --- | --- | --- | --- | --- | --- | --- | --- |
|  |  | **Hospital delivery** | **p** | **Timely initiation of antenatal care** | **p** | **Completion of five antenatal care visits** | **p** | **Timely completion of five antenatal care visits** | **p** |
| **Transportation subsidies for antenatal care visits** |  |  | 0.001 |  | 0.122 |  | 0.245 |  | 0.318 |
| No | 25(10.5) | 22(88.0) |  | 15(60.0) |  | 17(68.0) |  | 7(28.0) |  |
| Yes | 212(89.5) | 209(98.6) |  | 158(74.5) |  | 166(78.3) |  | 81(38.2) |  |
| **WeChat group chat** |  |  | 0.001 |  | 0.018 |  | 0.357 |  | 0.249 |
| No | 23(9.7) | 20(87.0) |  | 12(52.2) |  | 16(69.6) |  | 6(26.1) |  |
| Yes | 214(90.3) | 211(98.6) |  | 161(75.2) |  | 167(78.0) |  | 82(38.3) |  |
| **Browsing health education information** |  |  | 0.057 |  | 0.037 |  | 0.080 |  | 0.018 |
| No | 107(45.1) | 102(95.3) |  | 71(66.4) |  | 77(72.0) |  | 31(29.0) |  |
| Yes | 130(54.9) | 129(99.2) |  | 102(78.5) |  | 106(81.5) |  | 57(43.8) |  |
| **Understanding health education information** |  |  | 0.100 |  | 0.007 |  | 0.145 |  | 0.588 |
| No | 53(22.4) | 50(94.3) |  | 31(58.5) |  | 37(69.8) |  | 18(34.0) |  |
| Yes | 184(77.6) | 181(98.4) |  | 142(77.2) |  | 146(79.3) |  | 70(38.0) |  |
| **Interacting in the group chat** |  |  | 0.236 |  | 0.165 |  | 0.911 |  | 0.116 |
| No | 142(59.9) | 137(96.5) |  | 99(69.7) |  | 110(77.5) |  | 47(33.1) |  |
| Yes | 95(40.1) | 94(98.9) |  | 74(77.9) |  | 73(76.8) |  | 41(43.2) |  |

**Table S4 Associations between specific intervention methods and sociodemographic characteristics in Intervention group (n=237)**

|  | **Total** | **Delivery places** | | **Completion rate of antenatal care visits** | | | | | |
| --- | --- | --- | --- | --- | --- | --- | --- | --- | --- |
|  |  | **Hospital delivery** | **p** | **Timely initiation of antenatal care** | **p** | **Completion of five antenatal care visits** | **p** | **Timely completion of five antenatal care visits** | **p** |
| **Ethnicity** |  |  | 0.026 |  | 0.563 |  | 0.717 |  | 0.845 |
| Han | 6(2.5) | 5(83.3) |  | 5(83.3) |  | 5(83.3) |  | 2(33.3) |  |
| Yi | 231(97.5) | 226(97.8) |  | 168(72.7) |  | 178(77.1) |  | 86(37.2) |  |
| **Education** |  |  | 0.050 |  | 0.050 |  | 0.203 |  | 0.709 |
| Illiterate | 40(16.9) | 37(92.5) |  | 23(57.5) |  | 28(70.0) |  | 13(32.5) |  |
| Primary school | 138(58.2) | 137(99.3) |  | 104(75.4) |  | 105(76.1) |  | 51(37.0) |  |
| Junior high school or above | 59(24.9) | 57(96.6) |  | 46(78.0) |  | 50(84.7) |  | 24(40.7) |  |
| **Age** |  |  | 0.706 |  | 0.001 |  | 0.015 |  | 0.168 |
| <20 | 30(12.7) | 30(100.0) |  | 26(86.7) |  | 25(83.3) |  | 9(30.0) |  |
| 20-25 | 115(48.5) | 111(96.5) |  | 93(80.9) |  | 97(84.3) |  | 50(43.5) |  |
| 26-30 | 59(24.9) | 58(98.3) |  | 36(61.0) |  | 40(67.8) |  | 21(35.6) |  |
| >30 | 33(13.9) | 32(97.0) |  | 18(54.5) |  | 21(63.6) |  | 8(24.2) |  |
| **Annual household income** |  |  | 0.564 |  | 0.107 |  | 0.630 |  | 0.172 |
| ≤5000 RMB | 47(19.8) | 46(97.9) |  | 29(61.7) |  | 38(80.9) |  | 12(25.5) |  |
| 5001-10000 RMB | 118(49.8) | 116(98.3) |  | 87(73.7) |  | 92(78.0) |  | 46(39.0) |  |
| ≥10001 RMB | 72(30.4) | 69(95.8) |  | 57(79.2) |  | 53(73.6) |  | 30(41.7) |  |
| **Rural-urban migration experience** |  |  | <0.001 |  | 0.330 |  | 0.642 |  | 0.851 |
| No | 39(16.5) | 34(87.2) |  | 26(66.7) |  | 29(74.4) |  | 15(38.5) |  |
| Yes | 198(83.5) | 197(99.5) |  | 147(74.2) |  | 154(77.8) |  | 73(36.9) |  |
| **Parity** |  |  | 0.134 |  | <0.001 |  | 0.155 |  | 0.178 |
| 1 | 72(30.4) | 72(100.0) |  | 64(88.9) |  | 59(81.9) |  | 26(36.1) |  |
| 2 | 77(32.5) | 73(94.8) |  | 59(76.6) |  | 63(81.8) |  | 31(40.3) |  |
| 3 | 49(20.7) | 47(95.9) |  | 33(67.3) |  | 35(71.4) |  | 22(44.9) |  |
| ≥4 | 39(16.5) | 39(100.0) |  | 17(43.6) |  | 26(66.7) |  | 9(23.1) |  |
| **Walking time from home to the township hospital** | | | 0.001 |  | 0.274 |  | 0.661 |  | 0.939 |
| <30 min | 30(12.7) | 26(86.7) |  | 24(80.0) |  | 21(70.0) |  | 10(33.3) |  |
| 30-45min | 60(25.3) | 60(100.0) |  | 39(65.0) |  | 45(75.0) |  | 24(40.0) |  |
| 46-60min | 101(42.6) | 99(98.0) |  | 78(77.2) |  | 81(80.2) |  | 37(36.6) |  |
| >60 min | 46(19.4) | 46(100.0) |  | 32(69.6) |  | 36(78.3) |  | 17(37.0) |  |
